# The social production of substance abuse and HIV/HCV risk: an exploratory study of opioid-using immigrants from the former Soviet Union living in New York City

**DOI:** 10.1186/1747-597X-7-2

**Published:** 2012-01-12

**Authors:** Honoria Guarino, Sarah K Moore, Lisa A Marsch, Sal Florio

**Affiliations:** 1 Center for Technology and Health, National Development and Research Institutes, Inc., 71 W. 23rd St., 8th Fl., New York, NY, 10010, USA; 2 Center for Technology and Behavioral Health, Dartmouth Psychiatric Research Center, Department of Psychiatry, Dartmouth College, Rivermill Commercial Center, 85 Mechanic St., Ste. B4-1, Lebanon, NH 03766, USA; 3 Department of Behavioral Health, Chemical Dependency Services, Coney Island Hospital, 2201 Neptune Ave., Brooklyn, NY, 11224, USA

**Keywords:** Former Soviet Union immigrants, opioid use, injection drug use, HIV risk, HCV risk, qualitative methods

## Abstract

**Background:**

Several former Soviet countries have witnessed the rapid emergence of major epidemics of injection drug use (IDU) and associated HIV/HCV, suggesting that immigrants from the former Soviet Union (FSU) may be at heightened risk for similar problems. This exploratory study examines substance use patterns among the understudied population of opioid-using FSU immigrants in the U.S., as well as social contextual factors that may increase these immigrants' susceptibility to opioid abuse and HIV/HCV infection.

**Methods:**

In-depth interviews were conducted with 10 FSU immigrants living in New York City who initiated opioid use in adolescence or young adulthood, and with 6 drug treatment providers working with this population. Informed by a grounded theory approach, interview transcripts were inductively coded and analyzed to identify key themes.

**Results:**

The "trauma" of the immigration/acculturation experience was emphasized by participants as playing a critical role in motivating opioid use. Interview data suggest that substance use patterns formed in the high-risk environment of the FSU may persist as behavioral norms within New York City FSU immigrant communities - including a predilection for heroin use among youth, a high prevalence of injection, and a tolerance for syringe sharing within substance-using peer networks. Multiple levels of social context may reproduce FSU immigrants' vulnerability to substance abuse and disease such as: peer-based interactional contexts in which participants typically used opioids; community workplace settings in which some participants were introduced to and obtained opioids; and cultural norms, with roots in Soviet-era social policies, stigmatizing substance abuse which may contribute to immigrants' reluctance to seek disease prevention and drug treatment services.

**Conclusion:**

Several behavioral and contextual factors appear to increase FSU immigrants' risk for opioid abuse, IDU and infectious disease. Further research on opioid-using FSU immigrants is warranted and may help prevent increases in HIV/HCV prevalence from occurring within these communities.

## Background

In the two decades since the dissolution of the Soviet Union, an unprecedented wave of immigrants from the former Soviet Union (FSU) have entered the United States. Between 2005-2009, approximately 995,000 individuals born in the FSU were living in the U.S [1]. Although the surge of FSU emigration that occurred in the mid-to-late 1990's has ebbed, substantial numbers of FSU immigrants continue to arrive in this country [2]. New York City (NYC), the location of this study, has the largest concentration of FSU immigrants in the U.S. with more than 185,000 FSU-born residents, the majority of whom originated from Russia, Ukraine and Uzbekistan [1].

Immigrants from the vast, multi-ethnic territory of the FSU are a heterogeneous group with varied ethnic/racial identifications and religious affiliations - e.g., ethnic Russians, Central Asians, Jews and Christians. Yet they tend to see themselves as unified by their common history of living in the Soviet or former Soviet state, their shared use of the Russian language and a certain shared cultural identity [3,4]. Therefore, a focus on "FSU immigrants" or "Russian-speaking immigrants" has become a standard approach in research on this population.

Although FSU immigrants in the U.S. have been found to display certain sociodemographic characteristics generally thought to exert a protective effect on health, including supportive familial and community networks and high levels of education and marriage [5,6], studies have also documented elevated rates of disease such as diabetes and HCV in this population [7,8]. Thus, it appears as though FSU immigrants may represent an exception to the dominant "healthy migrant effect" [5] according to which lower levels of health-related risk behaviors, including substance use, have been observed among a variety of U.S.-based immigrant groups as compared to native-born populations [c.f. [9-11]].

Although precise statistics on substance use within Russian-speaking immigrant communities in the U.S. are lacking, as neither government data nor national surveillance studies track drug use or treatment admissions by national origin or non-Hispanic ethnicity, available evidence from multiple sources strongly suggests that the abuse of illicit substances - especially opioids - and injection drug use (IDU) have become growing problems within NYC FSU immigrant communities since the mid-1990's, particularly among adolescents and young adults. In addition to brief references in the substance abuse literature [12,13], this phenomenon is evidenced in recent articles in the popular press [14] and by the authors' personal communication with NYC substance abuse treatment providers and researchers. In a comparative profile of FSU-born substance users in Israel, Germany and the U.S., Isralowitz and colleagues [15] assert that "rates of alcohol and drug problems among [FSU] immigrants appear to be disproportionally high" in comparison to other immigrant groups and "possibly even native populations." Yet in spite of evidence suggesting high rates of opioid abuse and IDU among younger members of NYC's Russian-speaking immigrant communities, research on substance use among U.S.-based immigrants from the FSU has been surprisingly scarce.

We are aware of only two published reports on this topic, neither of which specifically concerns opioid use or is based on primary data collected from Russian-speaking substance users themselves, relying instead on clinical observations or interviews with individuals familiar with this population. Kagan and Shafer [16] use historical and clinical information to construct an overview of the role and meanings of substance use - particularly alcohol use - in Russian, Soviet and FSU immigrant culture. Noting that Russia has one of the highest rates of alcoholism in the world, the authors explain that in pre-revolutionary Russia, "communal intoxication" was a culturally-endorsed ritual; heavy drinking was normalized as a vehicle for sociability and an emblem of masculinity. Compounding the effect of this cultural approbation of drinking, Kagan and Shafer assert, the lack of substance abuse education and prevention programs in both the FSU and Russian-speaking communities in the U.S. may predispose FSU immigrant youth to substance abuse problems, as these youth are often unaware of the dangerous consequences of substance use. Isralowitz, Straussner and Rosenblum [17] offer a preliminary account of substance use behavior and treatment service utilization among drug-using FSU immigrants in NYC, based on interviews with public officials and administrators. Their results indicate that FSU immigrants display culturally-specific patterns of substance use including age-related substance preferences, with older immigrants preferring alcohol, or a combination of heroin and alcohol, and younger immigrants favoring heroin, with injection as the preferred route of administration. In contrast to other young drug users, they claim that FSU immigrant youth often start their substance use trajectories by injecting heroin, avoiding intermediary drugs or routes of administration.

Somewhat more literature on substance use among FSU immigrants has emerged from Israel. This research demonstrates that FSU immigrants tend to have more problem substance use relative to native-born Israelis, including significantly higher rates of: lifetime alcohol, marijuana and stimulant use, and past 30-day binge drinking, hard liquor and ecstasy use, among male adolescents [18]; and heroin use by injection, HCV and HIV among adult heroin users [19]. FSU immigrants in the latter study were also found to be significantly younger on average than their Israeli-born counterparts. In another study characterizing the population of Russian-speaking immigrant heroin users in Israel, 97% of FSU-born respondents reported having a pre-existing heroin addiction at the time of immigration to Israel; respondents were 21.94 years on average at their first use of heroin, which 73% reported occurring in the company of friends [20].

An important reason substance use among FSU immigrants merits concern is the potential for the spread of HIV and similar infectious diseases within this underserved community. Since the collapse of the Soviet Union in 1991, Russia and several other countries of the FSU have witnessed the explosive emergence of major HIV and HCV epidemics fuelled by alarmingly high rates of substance abuse, IDU and risk behaviors such as syringe sharing [21-24]. HIV prevalence in Eastern Europe and Central Asia has nearly tripled since 2000, reaching an estimated total of 1.4 million infections in 2009 [25], making it the only region in the world where HIV prevalence continues to rise [26]. A constellation of socio-structural conditions has contributed to the rapid rise of IDU in the post-Soviet region including: the emergence of Afghanistan as the world's largest opium producer; the opening of new drug trafficking routes through Central and Eastern Europe [27,28]; a political climate characterized by "punitive and repressive policies toward drug use" [24]; and the failure of most former-Soviet governments, especially Russia, to provide effective prevention measures such as syringe exchange and opioid replacement treatment [29,30]. Because of this high risk environment in their countries of origin, in combination with the reportedly high prevalence of opioid abuse and IDU within their post-immigration communities, young FSU immigrants living in NYC may be particularly vulnerable to injection-mediated transmission of HIV/HCV.

Research conducted among other migrant groups has shown that IDUs migrating from an environment characterized by high-risk drug use may maintain risky substance use practices in their destination environment. Studies of injection-drug-using Puerto Rican migrants to NYC, for example, have demonstrated that migrants who had previously used drugs in Puerto Rico had higher levels of injection-related risk behaviors, including greater injection frequency, shooting gallery use and sharing of injection paraphernalia, relative to both Puerto Ricans who had not used drugs in Puerto Rico and other groups of IDUs in NYC [31,32]. Thus, persuasive evidence suggests that social norms structuring substance use and injection behavior established in a high-risk originating environment can endure within migrant groups after relocation to an environment of lower risk (i.e., one with less risky behavioral norms and more harm reduction resources).

The present exploratory, qualitative study of opioid-using FSU immigrants in NYC aims to elucidate contextual factors that may make these immigrants particularly vulnerable to opioid abuse and IDU, as well as specific substance use behaviors that may place them at risk for HIV/HCV. Particular attention is paid to how the immigration experience, cultural norms and values, and the institutional and interactional settings within which opioid use occurs may shape immigrants' patterns of opioid use. In light of the dearth of research on substance-using FSU immigrants in the U.S., a goal of this study was to collect formative data that may inform future research with this population.

Rhodes' concept of the "risk environment" [33-35] provides a useful framework for conceptualizing contextual influences on the substance use behavior and disease risk of FSU immigrants. This framework holds that multiple levels of social context interact to construct particular "risk environments": micro-level factors (e.g., social networks); meso-level factors (e.g., perceived group norms, institutional policies and practices) and macro-level factors (structural forces such as poverty, cultural beliefs and social stigma). More important than drawing lines separating levels of risk, however, is appreciating the fact that these elements of context are necessarily inseparable in lived experience, interacting in complex ways to produce differential disease risk for different social groups.

## Methods

### Participants

In-depth interviews were conducted with 10 FSU immigrants who initiated opioid use in their adolescence or young adulthood. The rationale for targeting individuals who began using opioids in their youth was based on prior research suggesting that opioid use within the NYC FSU community is primarily a youth phenomenon [17], and was intended to enable an exploration of the social contexts surrounding FSU youths' opioid use initiation. In addition, 6 drug treatment providers with extensive clinical experience with substance-using FSU immigrants were interviewed in order to access their expert insight into drug use patterns and drug treatment issues characteristic of FSU immigrants that may distinguish them from other groups of opioid users in NYC. (Treatment-related findings will be reported in a separate paper.)

Opioid-using participants were required to: self-identify as being of Russian or FSU descent; be current or former users of opioids; have begun using opioids in their youth (≤30); and have sufficient fluency in English to participate in an interview (none were excluded based on this criterion). Treatment provider participants were current or former clinical staff members of three substance abuse treatment programs in NYC with significant numbers of FSU immigrant clients. Four of these providers were immigrants from Russia. One individual interviewed as a former opioid user was also a staff member at a treatment program with a substantial FSU immigrant client population, while one participant interviewed as a treatment provider self-identified as former opioid user during the interview.

Opioid-using participants were recruited via a combination of strategies including: opportunistic sampling from a NYC methadone maintenance program (n = 2); targeted referrals from an outpatient substance abuse treatment program providing Russian-language services (n = 5) and an HIV outreach program for substance-using youth (n = 1); and direct advertising in local Russian-language community publications (n = 2). Names referenced in this paper are pseudonyms.

### Interviews

Study procedures were approved by the Institutional Review Boards of National Development and Research Institutes and Maimonides Hospital, and all participants provided informed consent prior to being interviewed. In order to promote an open and honest dialogue, the study's strict confidentiality protections were explained to all interviewees, while opioid-using participants recruited through treatment programs were specifically assured that the information they provided would not be shared with program staff. Semi-structured, audiotaped interviews (1-1.5 hours in length) were conducted in English by the first author, trained as an ethnographer. All opioid-using participants were interviewed individually, while treatment providers were interviewed either individually or, in the case of those who worked at the same facility, in small groups of two or three. A total of 13 interviews were conducted. All interviewees were compensated $35 for their time.

In the formative stages of this study, informal exploratory interviews were conducted with treatment professionals and substance abuse researchers in the NYC area with knowledge of the target population; initial interview guides were developed from these conversations. The interview format was designed to be flexible, consisting of open-ended questions arranged in a variable sequence, to allow interviewees to introduce or elaborate on topics of importance to them. Specific interview questions were derived from the research objectives, addressing: participants' personal drug use trajectories and experiences with opioid use - particularly in relation to their immigration histories; the social and interpersonal contexts of their opioid use; their attitudes and beliefs around opioids and injection; and potential risk behaviors. Interviews were transcribed verbatim by a consultant, the transcriptions checked for accuracy by the first author.

### Data Analysis

The data analysis was informed by a constructivist grounded theory approach [36,37] which aims to situate emergent understandings of social processes and systems of meaning within their social, historical and interactional contexts. The core of this method consists of an inductive process of coding textual data to identify key themes and patterns; theoretical interpretations emerge from a multi-faceted comparative analysis that progresses through successive levels of abstraction [38]. In order to enhance the reliability of the analysis, the first two authors, both experienced in qualitative analysis, jointly conducted the content-based coding of interview transcripts. (Because of the small size of the dataset, no software program was used to assist in the analysis.) The analysts independently reviewed and coded approximately one half of the collected transcripts in an iterative process, meeting for periodic consensus sessions to develop and refine the code list and to assess inter-coder reliability in the application of the codes to the data. The first author then used the final coding schema to code the remainder of the data set. Next, transcript passages illustrative of each of the thematic codes were repeatedly reviewed and compared (i.e., the "constant comparative method" [39]) with particular attention paid to the most commonly voiced themes as well as inconsistencies among interviewees' accounts. The inclusion of two distinct participant subsamples also allowed for the triangulation of data from different sources, a technique commonly used in qualitative research to increase the validity and richness of findings [40]. Overall, the most prominent themes that emerged from the dataset were voiced by both opioid-using and treatment provider participants; findings specific to only one subsample are identified as such in the presentation of results.

## Results

### Characteristics of Opioid-using Participants

Among the 10 participants interviewed as opioid users, 5 were male; they ranged in age from 19 to 45 years old, with a mean age of 30.6 years. Four were married or living with a partner, 3 were divorced/separated and 3 were single. Four were currently employed. All were born in countries of the FSU: 4 in Russia, 3 in Ukraine, 2 in Kazakhstan and one in Uzbekistan. Three, all of Jewish heritage, immigrated to the U.S. prior to the Soviet Union's collapse in 1991; 6 immigrated between 1992-1999; one came in 2004. Most arrived in the U.S. in their adolescence (60%) or early childhood (30%), one in early adulthood. All but two participants initiated opioid use in the U.S. The majority (80%) identified heroin as their primary drug of choice, with the remaining two preferring either speedballs (heroin and cocaine combined) or methamphetamine and heroin equally. Seventy percent began using heroin as teenagers, 30% in their early-mid 20's. Notably, 100% used heroin via injection. Three participants who identified heroin as their drug of choice also regularly injected cocaine. Ninety percent had been in some form of substance abuse treatment, while 70% were currently in treatment.

Other participant characteristics reflect trends reported in the literature on FSU immigrants. Participants were generally well-educated, with 2 holding graduate/professional degrees and 4 others having attended at least some college. Four resided with their parents and most described relationships with parents and extended family as central to their lives. Two, however, were homeless. Ninety percent were bilingual in Russian and English (one no longer spoke Russian), an attribute which participants reported to be typical of the younger generation of FSU immigrants.

### Patterns of Opioid Use among Substance-using FSU Immigrants

#### 'What's your drug of choice? Heroin? Oh, you're Russian?'

It is the unanimous perception of participants, both opioid users and treatment providers alike, that the use of opioids, particularly heroin, is widespread among younger members of NYC's Russian-speaking immigrant community. Participants aver that among "Russians... dope is very popular.... more popular than any other drug." In Viktor's experience, Russians' predilection for heroin is so well-known among the City's substance users as to be commonplace knowledge: "almost like all the people that I've met in the treatment programs - 'Oh, what's your drug of choice? Heroin? Oh, you're Russian?'" (Following participants' own usage, the term "Russian" is used herein to refer broadly to individuals from countries of the former Soviet Union.)

Six respondents provided historical explanations for this propensity, noting that, while most illicit drugs were not readily available in the Soviet era, opium products, typically prepared at home from poppies, were in fairly widespread use: "Russians that come from the Soviet Union... [many] of them already are opiate-addicted. Because in my country, we didn't have cocaine... but we had a lot of poppies or poppy fields." (Pavel) Natalia explains that "a lot of" the immigrants who arrived in the U.S. with pre-established addictions to opium "were running from Russia to America to stop using... but once they heard that there's such a thing as heroin... they would switch to heroin." The substance use knowledge and practices of this early wave of immigrants then diffused to a younger generation; as Ivan puts it, older users who, in the Soviet Union, had used "morphine from hospitals, poppy in the kitchen," after immigrating to the U.S. "they shared the secrets. And the young people listened."

While opioid use is uniformly understood by participants to be a problem of the younger generation within the Russian community, heavy drinking is viewed as "old school", an affliction and emblem of one's parents and grandparents that is culturally accepted, especially for men. Although some participants had used alcohol in the past, they were consistent in expressing their strong preference for opioids over alcohol. Four reported paternal histories of alcoholism, with two proffering this as their primary motivation for intentionally avoiding alcohol. As Vladimir states, "My father, he drink all his life. I think he actually made me probably [an addict], and my grandfather died with vodka in his hand."

According to participants, opioid use patterns within the FSU immigrant community appear to be undergoing a shift, with the abuse of particular classes of opioids socially patterned by age cohort. Although there was relatively little abuse of opioids other than heroin within the present sample (only one participant - Viktor, the youngest at age 19 - reported significant abuse of prescription opioids), participants pointed out that illicit use of opioid analgesics is becoming increasingly widespread among teenage members of the FSU immigrant community. Vladimir attests that "I see... sixteen-, seventeen-year old kids... now they all have OxyContin, Percocet, everybody has in their pocket like change." This reported increase in the prevalence of prescription opioid abuse among FSU immigrants who are now adolescents is unsurprising as it mirrors the recent rise of prescription opioid abuse in the U.S. generally, particularly among youth [41].

Although it has been reported [17] that FSU immigrant opioid users in the U.S. tend to initiate their drug use careers with opioids, avoiding so-called "gateway" drugs, most participants in the present sample do not conform to this pattern. The majority (70%) initiated heroin use only after cycling through a succession of other drugs. Mikhail reflects that substance use was "like a ladder for me. First cigarettes, weed, and then I started doing cocaine" before ultimately progressing to heroin. This pattern is reversed with the two participants who began using drugs in the FSU prior to immigrating to the U.S., one of whom injected heroin before trying any other illicit drug while the other initiated heroin use very early in her drug use career. Yet providers report that, for most young Russian speakers, at least those whose substance use trajectories began in NYC, "heroin is the last stop on the train, not the first stop."

#### "Russians just seem to shoot, not sniff"

The uniform preference for injection heroin use in the present sample of opioid users corresponds to participants' perceptions regarding the wider NYC FSU immigrant community; they assert that injection is the predominant route of administration among Russian-speaking heroin users. According to treatment providers, a distinctly high prevalence of injection distinguishes FSU immigrants from other groups of heroin users in NYC.

Although two participants initiated heroin use via injection (one in Moscow and one in NYC), the majority of respondents began using the drug intranasally, progressing to injection after a period of time ranging from a couple days to three years. While treatment providers in the sample claim that "rapid" progression to injection is characteristic of young Russian-speaking heroin users, as has been reported by Isralowitz and colleagues [17], present data reveal a fair amount of individual variation. Nevertheless, four respondents did attest to remarkably brief periods of intranasal (or, in one case, inhalation) administration of heroin prior to transitioning to injection: Natalia, like Pavel, "started shooting up very quickly... within... a couple of months"; Sergei began injecting "after a week or two" of intranasal use; and Alexandra (whose opioid use began in the FSU) smoked heroin on only two occasions before injecting the drug.

Participants' individual reasons for favoring injection reflect those documented for other groups of heroin injectors [42-44], and include the perceived cost-effectiveness of injection as one's drug tolerance increases and the incomparable rush obtained from injecting heroin. Yet respondents also reported that injecting behavior has particular antecedents in Soviet and post-Soviet culture that may help explain FSU immigrants' distinctively strong preference for this route of administration. Several participants located the genesis of this preference in the Soviet era, when the primary psychoactive substances available, apart from alcohol, were homemade poppy-based formulas which were typically prepared in liquid form and thus had to be injected. As Igor, a Russian-born treatment provider, explained:

"Because it's homemade opiates, they started to inject it. Because [they] cannot sniff homemade liquids. They cannot drink it... there's only one way... they started injecting in Russia, and they come here with now the habit of injecting... now they're ready to continue injecting heroin. That's why a lot of Russians just seem to shoot, not sniff."

Thus, out of necessity, specific behavioral standards normalizing injection may have been forged among Russian that were then carried over into FSU immigrant communities.

Three interviewees advanced the possibility that the commonality of injection in routine Russian medical practice may function to legitimize injection among users of illicit opioids both in Russia and in the Russian immigrant community. Igor explains that, in Russian culture, not only is injection viewed as the safest and most effective mode of ingesting medications, "if you're afraid to [get an] injection, you're a coward. You have to be strong." Ivan further speculates that because blood donation was ennobled as "a part of [one's] social duties" in the Soviet era, some addicts developed "a very good relationship with [the] syringe." As a result of these sociocultural factors, in "the Russian drug addict community", there is "not such a big stigma of shooting... shooting is more acceptable." (Igor)

### Social Contexts Structuring Opioid Use within the FSU Immigrant Community

#### Cultural norms and values: Stigmatization of substance use within FSU immigrant culture

The reported cultural acceptance of injection does not extend to substance use or substance users, however; participants emphasized that an extreme stigmatization of drug users exists within Russian and FSU immigrant culture which stands in marked contrast to the acceptance of heavy drinking. According to these dominant cultural mores, drug use, especially heroin use, invokes multiple shames, as morally reprobate and déclassé: "my family was always telling me drugs is like it's the bottom... you can't have it worse than that" (Pavel). As Natalia elaborates, within this normative value system, drug addicts are viewed as both socially irredeemable: "Basically, once you have a habit... you're a lost cause to society... don't waste your time on them" and contaminated: "old-school Russians, they'll look at drug addicts as garbage... like people with leprosy, you don't want to go near them." Natalia traces the roots of this stigmatization to the treatment of addicts in Soviet and post-Soviet society: "As I was leaving Russia [in 1995]... there were no treatment centers at all. Drug addicts were not treated by anyone and no one wanted to deal with them, so it was like they usually get locked up with crazy people."

Yet, despite - or because of - the heavy stigmatization of drug use within mainstream Russian/FSU immigrant culture, among some FSU immigrants injection heroin use in particular has reportedly begun to accrue a counter-normative, 'outlaw' appeal. Ivan animates this attitude thusly: "They [are] proud to be addicts[s]. It's cool. 'I'm on the needle... I'm cool. Not like you, your life is so boring. My life is cool.'" Likewise, a Russian-born clinical staff member asserts that "when they use heroin, they are very proud... 'I don't drink, I don't do coke. I do heroin. That's a strong drug and I can handle that.'"

One potential ramification of this stigmatization of substance use may be reluctance within the immigrant community to openly discuss and educate youth about illicit drugs and addiction. Participants consistently disavow having had any substantive knowledge of opioids or the addiction process prior to becoming dependent. Marina's assertion that "I didn't know anything ... No information at all" is typical. Natalia implicitly links her ignorance about drugs to a moralization of substance use in which "good," middle-class people stand in contrast to drug users: "My parents were very well-educated... my whole family is like a good family. So no one ever taught me about drugs."

For almost all participants, their ignorance regarding illicit drugs encompassed the potential for developing physical and psychological dependence with continued use of opioids, as well as the physiological symptoms of dependence. Because they lacked an understanding of the highly addictive nature of opioids relative to other substances, three interviewees recounted being unable to recognize what was happening when they first experienced withdrawal symptoms. As Marina vividly describes:

"It was my first withdrawals, and I really don't know what is going on. I thought I just got sick. So when... I felt really terrible, I called my boyfriend [who had become her primary using partner] and I explained to him what is going on, and he just said, 'Listen, welcome to the club.' And I didn't understand. 'What do you mean, welcome to the club?' He said, 'It seems, baby, you hooked up and... from now on you're going to need it just to keep going.'"

The stigmatization of drug use within FSU immigrant culture may also complicate immigrants' harm reduction efforts, as the desire to protect oneself from disease was described as existing in tension with fear of community exposure as a drug addict. A Russian-born treatment provider explains how, for FSU immigrants, particularly youth, presenting oneself to a pharmacy in one's own neighborhood in order to purchase syringes can be fraught with the risk of social shame:

"It's too tight community. If I go to pharmacy to [buy syringes], everybody gonna know it. My relatives could come to the same pharmacy and pharmacist not going to keep my confidentiality. Not in Russian culture [laughs]. He or she definitely going to tell my mother that I do it."

As a result, other, more covert, channels for accessing syringes may be preferred; in addition to secondary exchange from network members, several interviewees reported drug dealers as their primary source of syringes and other injection paraphernalia. Although putatively sterile, the provenance of this dealer-sourced injection equipment is unverifiable.

#### The "trauma" of the immigration experience: "This is the best medicine"

Another key reason for the appeal of opioids underscored throughout respondents' narratives is the drugs' unparalleled ability to alleviate stress - in particular, the stress of the immigration experience.

Participants uniformly described opioids as psychic armor, a powerful means for coping with the upheaval and dislocation of immigration which most characterized as stressful, or even "traumatic" and "scary". Marina, who emigrated from Moscow at age 30 (at a significantly older age than most participants), describes her feelings upon her arrival in a well-known Russian neighborhood in Brooklyn:

"Actually it was shock for me because in my mind, America was something special. It was like a dream, dream country, and especially New York City... So when I got here, the first place I saw in Brooklyn was Brighton Beach, and I was *shocked*. It was *dirty*, a lot of garbage, the bad smell... And this is the first time in my life when I got really scared... .it was hard for me because I didn't know the English... I was depressed, I wasn't sure about my future, I wasn't sure if I made mistake to come in this country."

For these interviewees, opioids initially seemed to provide a benign "emotional crutch", in Anna's words - an anodyne to relieve the pressure of trying to assimilate into a new culture and the fear and loneliness that frequently accompanies these struggles. In Vladimir's view: "I think [heroin] is so popular [in the Russian community] because everybody... came to the new country, they have so much anxiety and this is the best medicine, a lot of people find." Vladimir who, by age 21 was working a high-pressure job on Wall Street with a wife and two children to support, describes his initial experience with heroin as a welcome respite from the pressure to succeed: "It was unbelievable... Nothing I ever felt in my life, that's how I felt... Depression gone, no feelings, I was numb, no work, no wife, no job, no money, nothing existed for seven hours."

For the majority of participants who immigrated to the U.S. during their adolescence, the stresses of immigration were compounded by the developmental pressures that are often emblematic of the teenage years. For these youth, opioids were commonly used to fit in with teenage peers and to camouflage their identity as outsiders in America. "Me moving to the United States being sixteen and like having to dive in into a different culture, different language, different values, different beliefs, different mentality, different everything... I know most of the peers I was with... We [used heroin] to kind of like cope and deal and blend in and like kind of like survive the teenage years in one piece." (Natalia); "I just wanted to... not feel like a white bird in between black birds. I just wanted to be accepted... " (Pavel) An additional strain felt by many of these young immigrants was the burden of parental expectation, a pressure to succeed in America that several participants reported was exerted on them by their families. Pavel reflects that his now-deceased mother made her hopes for him explicit - "I remember my mother [on] the plane... she told me, 'This is your new beginning, new country, new people. Make the best of it.'" - and he rues the fact that, in his mind, he disappointed her expectations through his years of opioid addiction and failed treatment attempts.

#### Interactional contexts of opioid use: The role of peer networks and romantic partnerships

One of the most robust findings in the present data concerns the specific interactional context in which participants' opioid use initiation and early-stage use occurred; nine respondents began using opioids in the context of peer groups comprised of fellow FSU immigrant youth - small circles of close friends, sometimes including relatives such as cousins and romantic partners as well. For some of these interviewees, witnessing members of their peer group using opioids sparked their curiosity, as they wanted to experience the high their friends were enjoying. Others, like Olga, began using heroin to avoid standing out as different from her circle of teenage friends: "I see everybody's using so... I started using like everybody." In other cases, peers exerted persuasion, even manipulation, to get their friends to try the drug. Mikhail's cousin, who was also his "best friend" and who had already become dependent on heroin, dared him to use the drug for a week and not become dependent. "Ever since then," Mikhail says, "I just, I couldn't stop"; for years thereafter this cousin, along with another close Russian friend, became Mikhail's primary using partners. Within this peer-based interactional context, the use of opioids thus becomes a shared ritual that seemingly functions to enhance interpersonal identification and facilitate bonding with one's Russian-speaking peer group.

The peer group is also a key source of norms regulating youths' substance-use behavior. Reflecting on his teenage years, Pavel explains that: "All my friends... normal guys that I used to just chill with, I see they do it and it's like, well, nothing happened to them... I see the guy's normal... he's got a car, he's living the good life. If he can try it, then... why not me?" Seeing his friends using heroin yet seeming to thrive normalized the behavior for him and reinforced the drug's seductive aura. Viktor captures the way in which opioid use can radiate through a peer network via behavioral modeling: "The certain people that I hung out with, that's when like a wave came. Like as soon as like one of them would try it, like everybody would do it." Thus, peer network connections appear to play a central role in producing and reproducing young FSU immigrants' vulnerability to opioid abuse. It should be noted that, while the social setting of the FSU immigrant peer group dominated interviewees' accounts of the early stages of their opioid use, as these individuals' drug use careers became more established and their dependence increased, there was a concomitant tendency for opioid use to become an increasingly solitary ritual and isolating experience.

Four of the five women in the sample also identified relationships with opioid-using, FSU-born boyfriends or husbands as a salient micro-contextual factor propelling the escalation of their heroin use. In Natalia's case, her boyfriend was a member of the FSU peer network in which she was introduced to heroin. Although three other women began using heroin with a group of platonic Russian-speaking friends, their use intensified to dependence in the context of an emotionally intense - and ultimately, socially isolating - romantic partnership.

For the vast majority of participants, their initiation to IDU occurred within a similar social context as did their initiation to opioid use, with the influence of the FSU immigrant peer network playing a critical role in their transition to injection. Pavel reflects that although he "would never [have] pick[ed] up a needle by myself," being embedded in a circle of opioid-using peers, most of whom were a few years older and had already progressed to injecting heroin, served to normalize this route of administration for him.

Respondents acknowledged that sharing of injection equipment is not uncommon among their FSU immigrant peers, particularly in exigent circumstances. In Pavel's experience, "[sharing] happened a lot... because there was a lot of circumstances... where we had the drugs, but there was no needles." As has been documented with other groups of IDUs [45], many interviewees explained that while they generally tried to avoid sharing, they were sometimes compelled to do so by extreme levels of physical discomfort and by environmental constraints: "When you're on cravings, you don't really [care] so much. You just need to feel better and... if it's late or something and the pharmacy's closed, I mean everybody... shares needles." (Sergei)

Several interviewees who initially disavowed sharing syringes clarified that they only share with certain people - namely, those with whom they regularly interact and have strong emotional bonds, such as close friends and romantic partners. Highlighting the logistical complications that can promote sharing within a peer group of injectors, Alexandra states, "It was like we were like a group of friends and we always used together, and then we did [share]... sometimes 'cause [our syringes got] mixed and we didn't know which one is whose." According to Mikhail, he and his small network of good friends felt safe sharing with each other: "we would share... but we knew that none of us had anything... " Marina and Olga each noted that they only shared syringes with their current or former boyfriends. These accounts suggest that among FSU injectors different social norms may regulate the sharing of injection equipment with strangers or casual acquaintances, on the one hand, and with intimates, on the other hand, such that the general prohibition against sharing is relaxed within the context of close peer-based groups and romantic relationships. The emotional closeness and trust that characterize these relationships may provide injectors with a false sense of security regarding their risk of HIV/HCV transmission. As if to underscore this point, Marina and Olga volunteered that they strongly suspect they contracted HCV from the boyfriends with whom they had shared injection equipment.

#### Institutional contexts of opioid use: The role of community establishments

In relating the stories of their initiation into opioid use and injection, four interviewees highlighted the pivotal role played by local businesses in FSU immigrant neighborhoods. These community-based establishments cater to a Russian-speaking immigrant clientele and routinely hire fellow community members for low-skill, low-wage positions - particularly youth with limited work experience and recent immigrants who, if they lack facility in English, may be unable to obtain employment outside the community. These institutional settings provide opportunities for recent immigrants and youth to form social connections with fellow FSU immigrants and, in so doing, may simultaneously function as sites in which immigrants are initiated into opioid use. According to Natalia:

"Every unqualified job that you can possibly have in terms of moving companies, Russian waiters or busboys, dancers, escort service, any type of construction... they all like are shot through with drugs... My ex-husband had a moving company, ninety-five percent of people that worked for him were Russians and ninety-five percent of those people were heroin addicts."

Viktor directly attributes his introduction to heroin and the rapid escalation of his use to the succession of Russian restaurants in Brooklyn in which he worked starting at age 15. He describes these restaurants as locations in which not only is heroin (as well as prescription opioid and cocaine) use widespread among staff, but: "There's [drug dealers] that come to the restaurants [and] the owners wouldn't dare say anything, like they know what goes on and they know how fat their pockets are. Like business goes well for them when they come."

Viktor narrates a life story in which his substance use trajectory paralleled his employment history; as he progressed from busboy to runner to waiter at a series of ever-swankier restaurants and supper clubs in Brooklyn, so too did his heroin use progress from occasional sniffing with a few close friends and co-workers to daily use and ultimately to solitary injecting in the stairwell of his mother's apartment building. Underscoring the key role the Russian restaurant environment played in sustaining his opioid use, Viktor states, "as soon as I got out [of treatment] and got involved in the same circle, that's it. As soon as I would... go back to the same job, the same environment, I'd start using again in a matter of days."

Although Vladimir had already been sniffing heroin for three years by the time he lost his Wall Street job, his new-found work as a driver for an escort service which employed a number of Russian women as escorts served as a convenient source of heroin and provided his entrée into injection. When asked how he began injecting, he explains:

"I found one girl who was from Russia. I was driving escort - instead of Wall Street, of course I lost everything, so I was driving, escort service driver - and she just came from Russia. She knew me so she was shooting dope [in the car]. And she said, 'Why waste eight bags when you can do one?''"

Highlighting the catalytic role that involvement in sex work can play in abetting drug use for some young FSU women, two female interviewees described their work as strippers - work which, they reported, sometimes leads to informal paid sex with male customers - as pivotal to the initiation and escalation of both their substance use in general and their opioid use in particular. As Natalia explains: "We came to the United States and everyone was broke, and what happened to me as... a female teenager, as it happens with many women here, it's either prostitution or dancing, so I was a stripper for seven years... that's how I fell into drugs." According to Natalia and Marina, in the many large strip clubs in the greater NYC area employing stables of FSU immigrant women as dancers, drug use and drug selling are rampant. Marina explains that it was difficult to avoid drugs in this environment: "a lot of customers... used to bring the stuff in the club and share it with the girls... [and] in every club, there was a man who sell this stuff, whatever you want." In addition, Marina felt she needed to use some psychoactive substance in order to cope with the emotional depredations of "danc[ing] almost naked." She started with alcohol, progressed quickly to cocaine, but soon found her drug of choice in heroin; after trying heroin for the first time, she "thought this is my best friend now."

#### "This is something you can really do things on"

The importance of certain workplace contexts in fueling participants' opioid use helps elucidate a pervasive theme in these interviews - namely, that a central part of heroin's appeal lies in the sense of being "in control" a user may maintain while under the drug's influence. Relative to alcohol and other drugs, participants reported, opioids allow one to remain able to function and work without being obviously impaired. As Pavel puts it, "[Heroin] is something that you could really do things on it, not be like running away from your shadow." For many, heroin initially seemed to "kill the pain," helping them to endure exhausting or degrading work. In Viktor's portrayal, heroin is widely viewed by Russian restaurant staff as a work aide; he recalls the co-worker who introduced him to heroin stating: "Oh, this is the only way that I'm able to work." Moreover, Natalia points out that, in contrast to alcohol, it is easier to conceal one's use of heroin from others - perhaps a critical factor within a culture that heavily stigmatizes substance use: "With heroin, it's one of the drugs that is not very visible when you use it... you can hide and you can camouflage, and it kind of gives you that protection... " Yet, for almost all interviewees, this sense of "control" proved to be illusory, as their dependence ultimately grew beyond their ability to manage.

## Discussion

These findings suggest that a distinct set of sociocultural, historical and behavioral factors coalesce within NYC's FSU immigrant community to comprise a particular risk environment that may be conducive to opioid abuse and IDU and may facilitate the spread of HIV and similar diseases. Turning first to the issue of why opioid use appears to be prevalent among younger Russian-speaking immigrants in NYC, present findings point to multiple socio-historical factors which may increase these immigrants' susceptibility to opioid abuse. One likely factor influencing this trend is the long-standing tradition of opioid use within the Soviet Union [27,28,46]. According to participants, opioid use was introduced into NYC FSU communities by early immigrants who arrived in the U.S. with pre-existing addictions; from these early arrivals, the practice gradually diffused to other community members. A key finding of the present research which aligns with this community-diffusion explanation is that the majority of participants initiated both substance use in general and opioid use in particular in NYC, not in the FSU. Not only does this finding contrast with prior research which has found that the vast majority of FSU immigrant heroin users in Israel arrived in their destination country already addicted [20,47], it also suggests that opioid abuse within NYC FSU immigrant communities is not limited to those individuals who established dependencies in their countries of origin. Because problematic opioid use appears to now be affecting community members who immigrated to the U.S. in childhood or adolescence, it is possible that the practice may in turn diffuse to second generation immigrants in the near future.

Although FSU immigrants share certain cultural traits (e.g., strong family ties, cultural norms that place high value on educational achievement) that are thought to exert a salutary effect on many immigrant groups, suppressing their rates of substance misuse to levels below those of U.S.-born individuals, present data, along with prior research, suggest that immigrants from the FSU may not conform to this "healthy migrant effect". It is possible that, for FSU immigrants, the effect of these hypothesized protective factors may, under certain conditions, be outweighed by the effect of an array of other contextual factors present in their sending and/or receiving environments that can function to increase their vulnerability to substance misuse and associated infectious disease.

In fact, many interviewees identified the "trauma" of the immigration/acculturation experience, trauma which appears to be exacerbated when immigration coincides with an individual's teenage years, as a motive force behind their opioid use; participants reported using opioids to assuage assimilation stress and to fit in with a new peer group. The influence of these peer networks may be another factor perpetuating FSU immigrants' susceptibility to opioid abuse. Interview data demonstrate that participants were typically initiated into opioid use and injection within social settings in the company of fellow Russian-speaking peers. While the peer context has been documented to play an important role in opioid use and injection initiation among a variety of substance-using groups, especially youth, [42,43,48], this micro-level social context may be particularly salient and have distinctive dynamics among FSU immigrant substance users. Participant testimony supports published evidence that communal opioid use within friendship networks has historical antecedents within Russian culture extending back to the Soviet-era.tradition of home-based opium preparation in which a group of individuals commonly pooled resources and gathered together to cook and consume the liquid product [27]. In the immigrant context, the apparently close linkage between opioid use and the FSU peer group suggests that the use of opioids functions not only to solidify peer network bonds, but perhaps also serves as a ritual of in-group membership, affirming FSU immigrants' sense of belonging to a cohesive ethno-cultural community in the U.S. even as their connectedness to their former cultural identity is challenged as a result of immigration. For women, intimate relationships with opioid-using FSU immigrant men emerged as an additional factor in the intensification of their opioid use, a dynamic that has been documented with other groups of drug-using women [49].

Interviewees' accounts indicate that certain establishments where young and/or recent FSU immigrants are likely to seek employment - including Russian restaurants and strip clubs - can also play an important role introducing some community members to opioids, functioning as nexuses of opioid distribution and use within the community and providing immigrant employees with ready access to drugs. Finally, FSU immigrants' specific beliefs and attitudes towards opioids may be another factor helping to sustain the prevalence of opioid abuse within this community. For this sample of participants, opioids were valued for their perceived ability to alleviate emotional distress while simultaneously being conducive to work.

Certain patterns of substance use reported by participants merit discussion as they complicate trends attested to in the (albeit limited) literature on FSU immigrant opioid users. First, the majority of interviewees did not initiate substance use with opioids, as has been reported to be characteristic of FSU immigrant addicts in NYC [17] but rather experimented with a succession of illicit substances prior to using heroin - a pattern documented with other groups of adolescent and young adult heroin users in the U.S. [48,50]. Interestingly, the two participants whose substance use trajectories began in the FSU pre-immigration are the only exceptions to this pattern within the sample; these two women used heroin either before using any other illicit drug or very shortly after initiating the use of illicit substances. Is it possible that the use of "gateway" drugs is a substance use pattern more typical of U.S. youth and that FSU immigrants who initiate substance use in the U.S. are acculturating to this locally dominant norm? Although present data are not sufficient to answer this question, we hope to explore this possibility in future research with larger samples.

Second, while Isralowitz and colleagues [17] have indicated that initiation of heroin use via injection seems to be the norm for FSU immigrants, this pattern was not observed within the current sample of participants, the majority of whom initiated heroin use intranasally, later progressing to injection. Recent research has shown that most young heroin users now initiate use of the drug via the intranasal route, with a proportion later transitioning to injection [12,48]. Of the three participants in this sample who either initiated heroin use via injection or progressed to injection almost immediately, two began their use of the drug in the FSU (the same two women cited above). Thus, another question to be addressed in future research is whether the initiation of heroin use via injection might be particularly characteristic of those individuals who began using the drug in the FSU. These potentially divergent patterns of substance use between immigrants who began using opioids in the FSU and those who started in the U.S. suggest that cultural and behavioral norms within an immigrant community are not uniform or static, but emerge from complex processes of acculturation, with countervailing pressures and influences. While the present data are preliminary, it may be that, for substance-using FSU immigrants, some endurance of drug use norms characteristic of the FSU context is combined with a certain degree of adaptation to drug use patterns that are more typical of substance users in the U.S..

Despite possible differences in substance use trajectories between those whose use began in the FSU and those who initiated use in the U.S., this study provides evidence that a general preference for injection may link FSU immigrant opioid users in NYC with their counterparts in the FSU. It has been well documented that injection is the predominant route of administration among heroin users in Russia and other FSU countries [22,24]. Similarly, all substance-using participants in this sample exhibited a strong preference for injection, while interviewees uniformly reported injection to be typical of FSU immigrant opioid users. Just as Rhodes et al. [51] attest that the "pre-existing culture of group" injection of homemade opium preparations within the Soviet Union facilitated the rapid diffusion of injection heroin use within post-Soviet society, so too could this cultural precedent be exerting a similar influence within FSU immigrant communities today.

FSU immigrants' reported propensity to administer opioids via injection puts them at clear risk for HIV/HCV. The fact that injection opioid use appears to be largely a youth phenomenon within the FSU immigrant community is also cause for concern; recent research indicates that adolescents may be at increased risk for a host of health-related harms as youth may be more likely to engage in risky behavior in comparison to adults [52]. Additionally, certain sociocultural factors may heighten infectious disease-related vulnerability in this population. On the macro-contextual level, the acute and pervasive stigma attached to drug users within Russian culture, rooted in Soviet-era social policies institutionalizing harsh treatment of addicts [16], along with youths' lack of knowledge regarding addiction, may contribute to reluctance to seek treatment and harm reduction services, which may in turn increase the risk for HIV/HCV transmission among substance-using FSU immigrants.

On the micro-contextual level, the peer-based setting in which participants' opioid use and injection commonly occurred also has important implications for HIV/HCV risk as this interactional context may promote the sharing of syringes and other injection paraphernalia. In an instrumental sense, injecting in close proximity to other injectors has been found to increase the likelihood that sharing will occur (whether deliberately or accidentally) if sterile equipment is unavailable [27,51]. Moreover, the social norms that regulate peer groups of opioid-using FSU immigrants appear to be relatively tolerant of syringe sharing, just as the bonds of trust that characterize these relationships may encourage sharing. These behavioral norms may again trace back to Soviet and post-Soviet traditions of communal substance use and injection, which is strongly associated with the *sharing *of injection equipment [27,51]. Present data support the provisional hypothesis that certain FSU-born individuals who immigrated to the U.S. with prior substance use histories carried with them normative drug use practices formed in the high-risk environments of their home countries, including a preference for injecting and tendency to share injection equipment. Then, via network ties with other Russian-speaking opioid users in the U.S., some of whom initiated substance use post-immigration, these risky norms may have diffused to a wider community of users.

## Limitations

Qualitative investigations such as this that do not employ a methodological apparatus including probability sampling and standardized measures are not intended to produce findings generalizable to a larger population; instead, they aim to provide fine-grained accounts of participants' lived experiences and systems of meaning from their own perspectives. Nonetheless, certain limitations of this study qualify the present findings, foremost among which is the small sample size. The sample is also heavily weighted with treatment-experienced participants. Further research with a larger and more diverse sample, including treatment-naïve and out-of-treatment individuals, would allow for comparative analyses of potential differences among opioid-using FSU immigrants. Another limitation is the retrospective nature of participants' accounts, particularly of their initiation to and early-stage opioid use; while all initiated opioid use in adolescence or early adulthood, most were interviewed some years later, raising issues of limited recall and possible distortions of memory. Despite these limitations, this exploratory research has collected novel data on an understudied population that may be of considerable interest to substance use and HIV prevention researchers and service providers.

## Conclusions

Multiple social processes and contexts that inform the lives of substance-using FSU immigrants - from the experience of trans-national immigration, to cultural traditions and behavioral norms structuring substance use in the FSU and the U.S., to institutional and interactional settings in which immigrants may be exposed to and initiate use of opioids - appear to increase their risk for opioid abuse, IDU and injection-mediated infectious disease. Evidence from this exploratory study suggests that, for these immigrants, the distinct risk environments of both their homeland and their destination country play fundamental roles in shaping substance use behavior and health. Further research on potential sources of vulnerability to opioid misuse and blood-borne disease in this population, including socio-structural risk factors, may contribute to the design of effective prevention interventions, and may help avert increases in HIV/HCV rates within these communities, potentially preventing larger-scale social problems.

These findings suggest a need to expand and tailor prevention and harm reduction services to serve FSU immigrants - most clearly, by incorporating Russian-language materials and services. Certain approaches to substance abuse and HIV/HCV prevention may be particularly appropriate for FSU immigrants, especially youth, and merit further investigation. There is a need for sophisticated substance abuse prevention efforts targeting youth in Russian-speaking immigrant communities - in particular, fine-grained education that makes distinctions among different classes of drugs of abuse and clearly delineates the psycho-physiological effects of opioids, underscoring their highly addictive quality. With regard to HIV/HCV prevention, peer-based intervention models may represent a promising approach for this population given evidence of the social contexts of their opioid use. Peer-based approaches are effective in part because of their demonstrated ability to penetrate social networks of users who are unlikely to access mainstream treatment [53]. Also, peers may be perceived as trustworthy sources of prevention messages and may therefore have particular legitimacy within "hard-to-reach" communities such as the Russian-speaking immigrant community.

## Competing interests

The authors declare that they have no competing interests.

## Authors' contributions

HG participated in the design of the study, conducted all interviews, supervised and co-conducted the data analysis, and drafted and revised the manuscript. SKM participated in the data analysis, commented on the original draft and contributed to the revision of the manuscript. LAM conceived of the study, participated in the study design, commented on the original draft and contributed to the revision of the manuscript. SF participated in the study design, commented on the original draft and contributed to the revision of the manuscript. All authors have approved the final manuscript.
